# Vascular Aging and Atherosclerosis: A Perspective on Aging

**DOI:** 10.14336/AD.2024.0201-1

**Published:** 2024-02-01

**Authors:** Shudong Ma, Xuena Xie, Rong Yuan, Qiqi Xin, Yu Miao, Sean Xiao Leng, Keji Chen, Weihong Cong

**Affiliations:** ^1^Faculty of Chinese Medicine, Macau University of Science and Technology, Macau, China.; ^2^School of Pharmacy, Macau University of Science and Technology, Macau, China.; ^3^Laboratory of Cardiovascular Diseases, Xiyuan Hospital, China Academy of Chinese Medical Sciences, Beijing, China.; ^4^National Clinical Research Center for Chinese Medicine Cardiology, Xiyuan Hospital, China Academy of Chinese Medical Sciences, Beijing, China.; ^5^Division of Geriatric Medicine and Gerontology, Department of Medicine, Johns Hopkins University School of Medicine, Baltimore, MD 21224, USA.

**Keywords:** vascular aging, aging, atherosclerosis, cell senescence, mechanism

## Abstract

Vascular aging (VA) is recognized as a pivotal factor in the development and progression of atherosclerosis (AS). Although various epidemiological and clinical research has demonstrated an intimate connection between aging and AS, the candidate mechanisms still require thorough examination. This review adopts an aging-centric perspective to deepen the comprehension of the intricate relationship between biological aging, vascular cell senescence, and AS. Various aging-related physiological factors influence the physical system's reactions, including oxygen radicals, inflammation, lipids, angiotensin II, mechanical forces, glucose levels, and insulin resistance. These factors cause endothelial dysfunction, barrier damage, sclerosis, and inflammation for VA and promote AS *via* distinct or shared pathways. Furthermore, the increase of senescent cells inside the vascular tissues, caused by genetic damage, dysregulation, secretome changes, and epigenetic modifications, might be the primary cause of VA.

## 1. Introduction

Similar to the rest of the human body, the vascular system ages over time. Aging is an important risk factor for atherosclerosis (AS) [1-3]. Some pathological changes such as intima-media thickness, plaque, calcification, intraplaque hemorrhage, and stenosis appear in arteries with aging [4-9]. Epidemiology and pathology studies have revealed that AS-related disease processes begin early in life [10-13]. Interestingly, as people get older, the correlation between risk factors such as high blood pressure, calcium density, and AS becomes increasingly pronounced [14-16]. Recent research indicates that, except for biological aging, epigenetic aging might accelerate the development of cardiovascular illnesses (CVDs), implying that epigenetic aging is connected with AS in different individuals at the same age level [17].

Most blood vessels consist of three layers: the tunica intima, tunica media, and tunica adventitia, which primarily rely on the presence of endothelial cells (ECs), smooth muscle cells (SMCs) and connective tissue. In a young state, vascular cells can grow, reproduce, migrate, and secrete regularly, which attributes are vital for maintaining structural integrity, appropriate tension, normal blood flow, barrier function, substance metabolism, and new structure formation. However, when vascular cells get senescence due to the constant effect of various stressors and environmental variables, they become vulnerable to injury and permanent cell cycle stoppage. Senescent cells build up in vascular tissues and suffer growth arrest while escaping programmed death. Meanwhile, the senescence-associated secretory phenotype (SASP) occurs during cellular senescence, where cells release a plethora of secretory factors that may activate senescence pathways and further age adjacent cells. These terms of events lead to vascular aging (VA) and expedite the development of AS through a sequence of structural and functional alterations [18].

**Figure 1.**
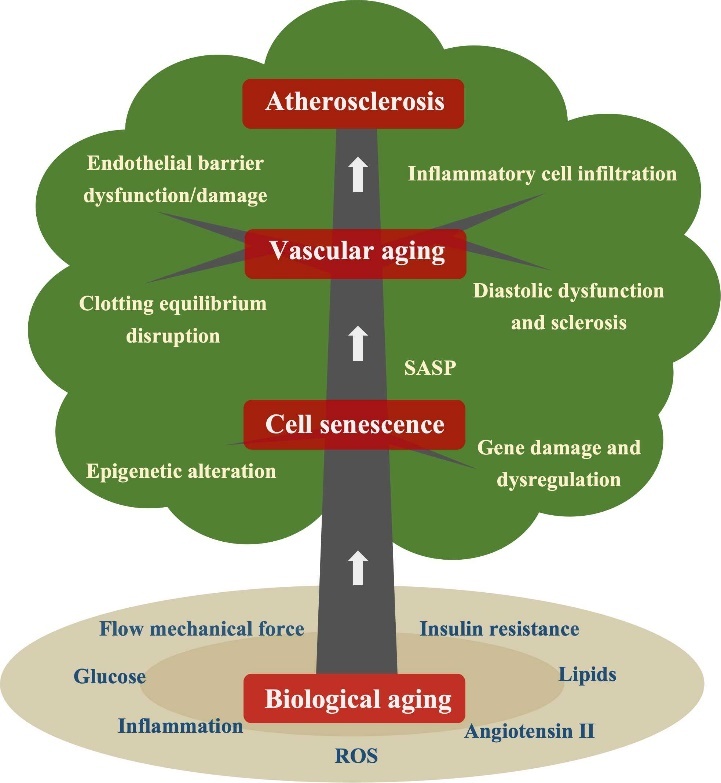
Vascular aging and atherosclerosis stem from biological aging and cell senescence.

In this paper, we present a comprehensive review of recent advancements in the understanding of the mechanisms underlying biological aging, vascular cell senescence, and their association with AS (Fig. 1)

## 2. Biological aging and AS

**Figure 2.**
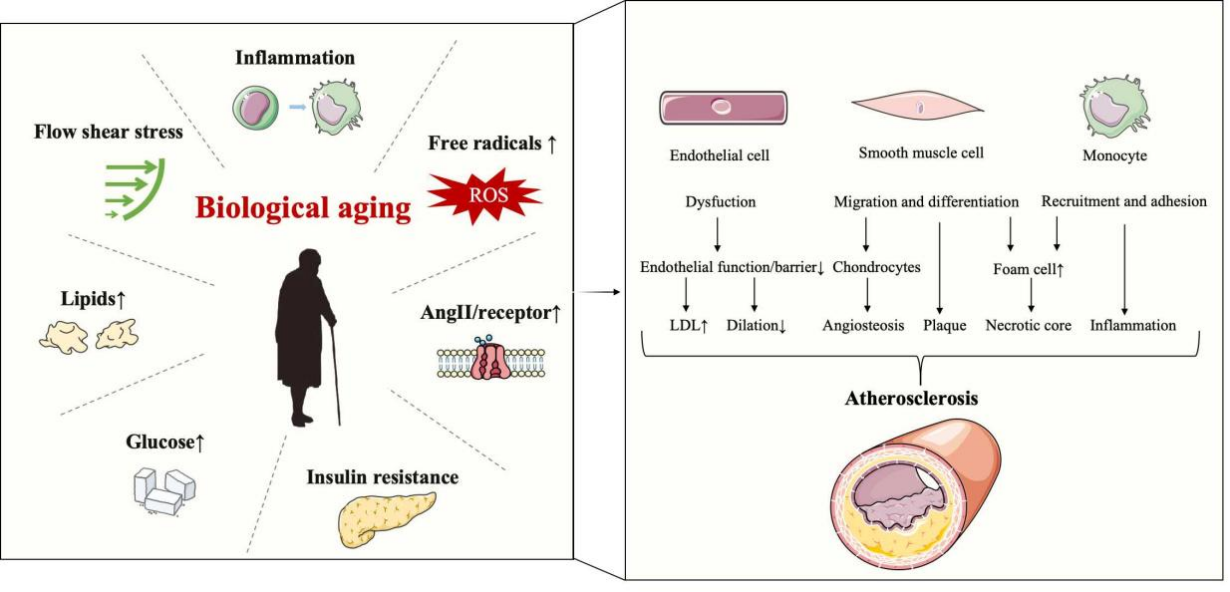
**Biological aging-related physiological variables impact different cells involved in the formation of AS**. EC dysfunction reduces vascular dilatation while increasing LDL transmembrane transport. The migration of activated SMCs, which can differentiate into foam cells and osteoblasts in response to lipid peroxides, is connected to plaque formation and vascular calcification. Monocytes can differentiate into macrophages and foam cells, contributing to vascular inflammation by recruitment and adhesion.

Biological aging influences many physiological variables, such as the accumulation of free radicals, inflammation, renin-angiotensin system excitation, blood flow mechanical force, and lipid and glucose metabolism anomalies. These stimuli alter the phenotype of ECs, SMCs, and monocytes, resulting in local vascular inflammation, calcification, endothelial dysfunction, low-density lipoprotein (LDL) deposition, and the formation of atherosclerotic plaques (Fig. 2).

### 2.1 Free radical accumulation

Free radcals accumulation is a hallmark of aging [19-22]. The increase in production but the decrease in elimination of reactive oxygen species (ROS) help free radical accumulation and oxidative stress, further resulting in oxidative damage. Mitochondria are known as the primary source of ROS generation [23], while antioxidant proteins in mitochondria may decline with age, such as aldehyde dehydrogenase-2 and manganese superoxide dismutase, to promote ROS production [24]. In addition, mitochondrial DNA (mtDNA) is particularly vulnerable to oxidative damage because of its proximity to the source of ROS production within the inner mitochondrial membrane and the absence of the protective histones [25], which may further increase ROS levels [24, 26]. With age, the activity of nicotinamide adenine dinucleotide phosphate (NADPH) oxidase rises to convert NADPH and oxygen to NADP^+^ and superoxide anion (O_2_^-^) [27, 28]. When O_2_^-^ diffuses from the mitochondria into the cytoplasm, it can be transformed into hydrogen peroxide (H_2_O_2_) by mitochondrial superoxide dismutase (SOD) [29]. The process gradually builds up oxidative damage, eventually leading to cell, tissue, and organism injury [30]. Moreover, ROS may act as signal molecules that impact cells within vascular walls, such as ECs, SMCs, and monocytes, which promote AS [31].

#### 2.1.1 ROS and endothelial dysfunction

Peroxynitrite (ONOO-) has a strong oxidative nitrifying effect, can induce DNA damage, mitochondrial dysfunction, oxidation of lipoproteins and protein nitroso, and increase the consumption of manganese superoxide dismutase. When superoxide anion enters the phospholipid membrane and interacts with endothelial nitric oxide (NO) synthase (eNOS), it produces ONOO-, which inhibits NO generation and limits NO-dependent vascular relaxation [24, 32]. In addition, ONOO- inhibits eNOS action by oxidizing its cofactor tetrahydrobiopterin (BH4) and consumes NO in this process [33]. These ROS may also initiate a vicious cycle of EC dysfunction by lowering NO bioavailability and activating NADPH oxidase superoxide [34], as well as further deactivating key enzymes involved in maintaining vascular homeostasis, such as prostacyclin synthase and cyclic guanosine monophosphate [35, 36].

#### 2.1.2 ROS and SMC activation

SMC is the primary source of extracellular matrix within plaques and fibrous caps, assisting in the production of extracellular matrix, glycoproteins, and proteoglycans [37, 38]. When SMCs are activated by ROS, they may migrate, differentiate, and get phenotypic alterations. For example, Salusin-B, a tiny protein molecule produced by the hypothalamus, triggers nicotinamide adenine dinucleotide phosphate oxidase 2 to produce O_2_^-^. This, in turn, stimulates SMC migration and neointima development by inhibiting NF-κB and blocking p65-NF-κB nuclear translocation [39]. In addition, under oxidative stress, SMCs may secrete cyclophilin A to promote their proliferation and contribute to the progression of AS [40]. Furthermore, H_2_O_2_ may modulate SMC synthetic phenotype [41]. For example, H_2_O_2_ can potentially increase Runx2 transcription, a key transcription factor in osteoblast and chondrocyte development, which causes osteogenic gene expression in SMC to ossification and angiosteosis [42]. Similarly, in response to H_2_O_2_, osteopontin (an adhesion molecule) is increased in SMCs, which collaborates with integrins to promote adhesion, migration, and apoptosis [43, 44]. Moreover, increased mortality in SMCs during aging, caused by reduced levels of superoxide dismutase 2, is related to the Act/FoxO3a pathway. The process contributes to sclerosis by causing vascular wall remodeling, inherent changes in SMCs rigidity [45].

#### 2.1.3 ROS and monocyte recruitment

Oxidation products activate mononuclear cells, initiating immune inflammatory responses and releasing chemotactic factors that attract other immune cells to injure vascular tissues, accelerating VA and plaque formation. For example, O_2_^-^ impede phosphatase 2A’s dephosphorylation of kappa B kinase protein inhibitors. The blockade causes the breakdown of IκBα (NF-κB inhibitor), then activates the transcription of NF-κB [46]. The IKK-NF-κB pathway promotes the synthesis of monocyte chemotactic protein-1, which is required for monocyte recruitment, adhesion, and chemotaxis [28, 47].

### 2.2 Lipids and ox-LDL

The plasma concentration of LDL increases with age [48, 49]. LDLs may penetrate the inner membrane when EC configuration and LDL permeability change [50]. ROS oxidizes LDLs and converts them into oxidized LDLs (ox-LDL) within the inner membrane, which is identified by the Lectin-like oxidized-low density lipoprotein receptor-1 (LOX-1) [51]. Ox-LDL can activate the mitogen-activated protein kinase (MAPK) and lead to the activation of particular cell adhesion molecules, such as monocyte chemoattractant protein 1 (MCP-1), vascular cell adhesion molecule 1 (VCAM1), and intercellular adhesion molecule 1 (ICAM1). Such activation promotes the formation of necrotic cores and plaques by facilitating monocyte recruitment and adherence [52, 53]. Furthermore, ox-LDL can induce ROS generation and activate the NF-κB pathway, leading to the secretion of adhesion factors [54, 55]. Ox-LDL has also been shown to stimulate phagocytosis in macrophages and SMCs [56-58]. For example, ox-LDL can be acted through mitochondrial reactive oxygen species to enhance the transcription activity of FBJ osteosarcoma oncogene to facilitate LOX-1 expression, increase lipid uptake and propel VSMC-derived foam cell formation and AS [59]. Research shows that SMCs, rather than macrophages, may be the primary source of foam cells in the vascular intima [60], demonstrating how the death and differentiation of SMCs connect with the formation and instability of plaques [61, 62].

### 2.3 Inflammation

Aging is recognized as a chronic, low-grade, systemic inflammatory state [63]. Inflammation is an essential biomarker of AS and is influenced by various variables such as oxidative stress, AngII activation, and insulin resistance [64, 65]. Inflammatory factors include TNF-α, C-reactive protein (CRP), NF-κB, interleukin-6 (IL-6), interleukin-18 (IL-18), and fibrinogen associated with age [65, 66]. TNF-α level, for example, is elevated with aging, which maintains the chronic proinflammatory vascular milieu and endothelial barrier damage *via* the VEGF/VEGF receptor-2 pathway [67]. Further, higher TNF-α levels cause platelet hyperreactivity, resulting in increased platelet procoagulant potential, blood adhesion, and thrombosis [68]. CRP induces the expression of the receptor for advanced glycation end-products in ECs, which plays a critical role in the development of AS by inducing ROS and activating the extracellular signal-regulated kinase/NF-κB pathway [69]. In addition, aging causes the release of inflammatory cytokines *via* the NF-κB-driven NADPH oxidase 1 (Nox1)-dependent pathway [70], which enhances the invasion of peripheral mononuclear cells and the buildup of macrophages in the vasculature [71].

### 2.4 Angiotensin II excitation

With aging, the AngII and its receptor may become more active [72-74]. Researchers found that ageing mice and monkeys show an enhanced Pr-Ace-AngII-AT1R axis but a compromised ACE2-MasR axis [72, 74]. In addition, autoantibodies targeting angiotensin II type 1 receptors were found definitively to cause artery inflammation and oxidative stress, hastening VA and AS. AngII can raise IL-18, activate the NF-κB pathway [75, 76], and increase MCP-1 expression, allowing monocytes to infiltrate vascular walls and leading to persistent vascular inflammation [77, 78]. Furthermore, AngII may increase the activities of CD40 and its ligand CD40L [79], which activates immune cells, cytokines, and other effector chemicals to cause the inflammatory milieu within the arteries. Moreover, AngII stimulates NADPH oxidase to produce O_2_^-^, which may be related to zinc distribution in mitochondria, increase the generation of ROS, and then cause oxidative damage in the arteries [80, 81].

### 2.5 Flow mechanical force

Alterations in the mechanical forces of blood flow within the vascular system are crucial physical signs of the aging process, particularly blood shear stress and blood pressure (BP).

#### 2.5.1 Unstable flow shear stress and turbulence

Shear stress in blood flow has a long-term and bidirectional effect on vascular walls [82]. The vasculature is protected by stable shear stress [83, 84], whereas unstable shear stress will affect the branches and sections that are vulnerable to turbulence [85]. This mechanical force potentially damages the endothelium and leads to EC dysfunction [86, 87]. As a result, plasma lipids are easier to permeate across ECs and build up in the vascular intima [50]. Furthermore, unstable force activates inflammatory pathways to oxidative stress response [84, 88-90], decreases NO bioavailability [91] and may induce EC to apoptosis [85, 92, 93], as well as causes SMC migration and differentiation [94]. In addition, excessive regional vascular remodeling during plaque formation aggravates unstable circumstances [95], resulting in fibre cap damage and thrombus formation [94].

#### 2.5.2 Hypertension and barrier disruption

Hypertension is commonly recognized as a significant risk factor for AS [96-98]. BP values regularly and continuously rise as people get old [99]. According to studies, arterial hypertension raises NADPH oxidase activity due to constant mechanical stress, which promotes ROS generation and MMP expression and then injures the vascular wall barriers, causing LDLs to stack up in the intima to develop AS [100-102].

### 2.6 Glucose and insulin resistance

Glucose levels and insulin resistance frequently elevate with advancing age [103, 104], attributed to low-level inflammation and lipid and nutrition metabolism issues [105-107]. Previous reports have clarified how plasma hyperglycemic circumstances cause AS [108]. The critical pathway is the activation of protein kinase C and its downstream pathway, which increases ROS and controls NO production. The process also triggers the release of endothelin 1 (ET-1), inducing vascular contraction and platelet aggregation [109]. In addition, ROS triggers the activation of NF-κB and downstream inflammatory genes in these processes, which causes monocyte adhesion, rolling, and shedding and eventually produces foam cells inside the subendothelial layer [110, 111].

Insulin resistance may promote endothelial dysfunction and accelerate AS by increasing the phosphoinositide 3-kinase/Akt pathway, which boosts ROS while lowering NO bioavailability and reduces susceptibility to prostacyclin [109, 112, 113]. In addition, at high cholesterol and sugar levels, NADPH oxidase was activated in monocytes to produce H_2_O_2_, which can promote the inflammatory motivation of monocytes in the vessel [114, 115].

## 3. Origens of vascular cell senescence and AS

**Figure 3.**
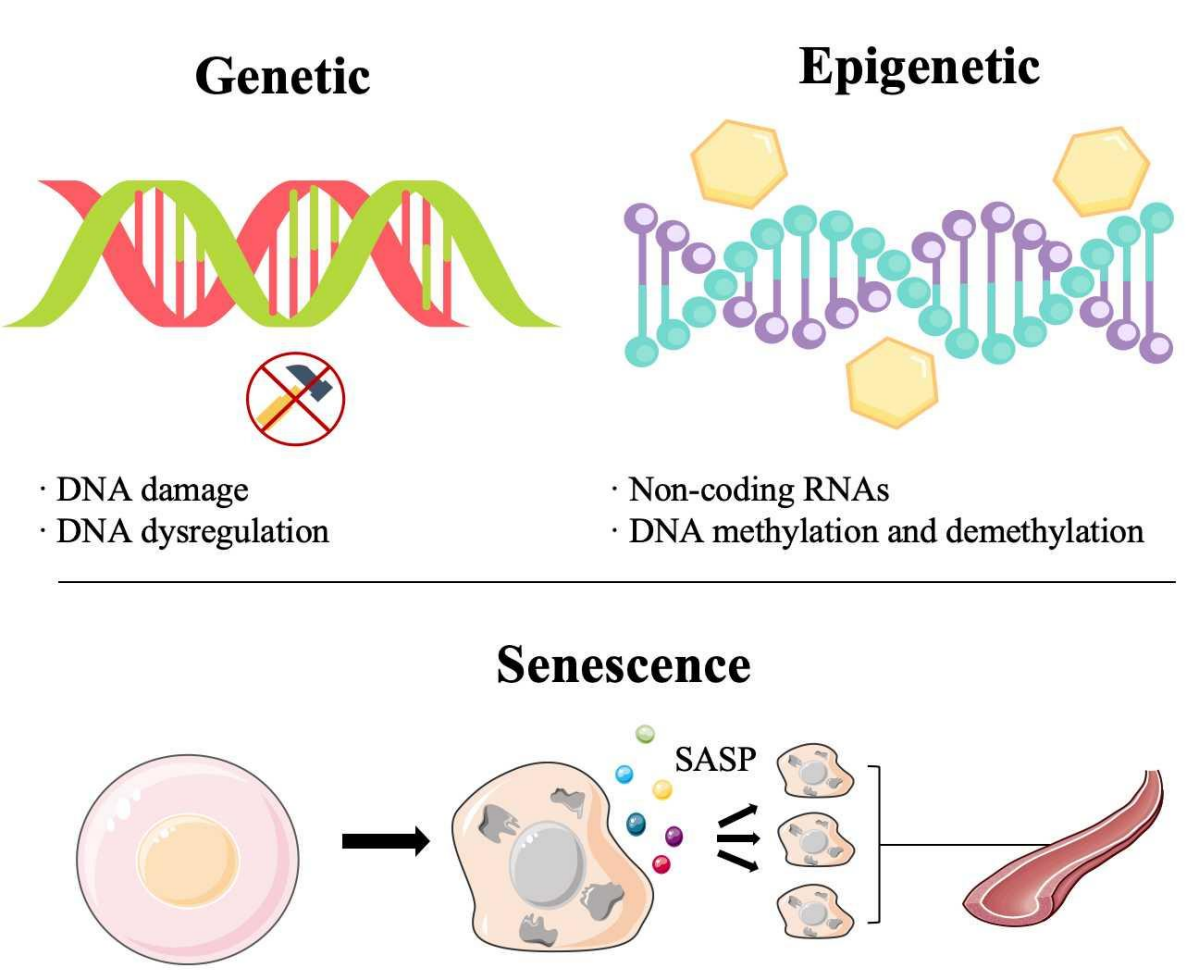
**Vascular cell senescence is traced to genetic changes such as DNA damage, dysregulation, and epigenetic alterations, including DNA methylation, demethylation, and noncoding RNA control**. Senescent cells produce SASP molecules, which act as signaling molecules transmitted between cells, thereby increasing the population of senescent cells inside vascular tissue.

Cell senescence is a state where the cell cycle is permanently stagnated. Strangely, despite their inability to proliferate, senescent cells retain metabolic activity and viability [116]. Cell senescence may be traced to DNA damage, DNA dysregulation, and epigenetic alterations, which cause changes in cells' secretomes. More senescent cells may build up in vascular tissues because the body's ability declines to eliminate them. Eventually, this process helps degeneration of the vasculature's intrinsic structure and function, potentially ending in the development of AS (Fig. 3).

### 3.1 Gene damage and dysregulation

#### 3.1.1 DNA damage

DNA damage and the obstacles in repairing after damage contribute to cell senescence [117, 118]. For example, researchers found that H_2_O_2_ or radiation-induced DNA damage triggers extracellular shuttling and downregulation of La ribonucleoprotein 7, reducing SIRT1 deacetylase activity to boost p53 and p65 transcriptional activity. The finding demonstrates that the activation of the ATM-LARP7-SIRT1-p53/p65 axis promotes cell senescence and AS [119]. In addition, nucleotide excision repair genes ERCC1 and XPD defective cause genomic instability, induce vascular cell senescence, and may further develop vasodilator dysfunction and increased vascular stiffness [120, 121]. Small nucleolar host gene-12 decreases in atherosclerotic lesions, which inhibits DNA-dependent protein kinase activity and impairs DNA repair capability. This process causes DNA damage response and cell senescence, which may implicate chronic vascular disease states and aging [122]. Furthermore, research found that mtDNA damage is associated with impaired proliferation and apoptosis of SMCs, as well as increased apoptosis and inflammatory cytokine release of monocytes [123].

#### 3.1.2 DNA dysregulation

DNA dysregulation, such as gene expression loss and deletion, may trigger cell senescence and AS. For example, age-related SIRT1 loss causes persistent DNA damage and DNA repair deficits, which further impair SMC reproductive capacity to reduce the fibrous cap thickness [124, 125]. Similarly, recent research found SIRT2 deficiency exacerbates age-related arterial stiffness and constriction-relaxation dysfunction, accompanied by aortic remodeling, vascular medial layer thickening, elastin fibre breakage, collagen deposition, and inflammation [126]. SIRT6 expression is reduced in human and mouse plaque VSMCs, which regulates senescence and osteogenic differentiation of VSMC to inhibit atherogenesis [127, 128]. In addition, atg7, a particular autophagy gene, loss in SMCs accelerates AS [129]. Also, the TET2 gene is absent in geriatric patients and induces NLRP3 activation to raise interleukin-1β levels, further causing atherosclerotic alterations [130]. Moreover, deletion of peroxisome proliferator-activated receptor coactivator-1 (PGC-1) reduces the production and activity of telomere reverse transcriptase, which may lead to DNA damage and accelerate cell senescence and AS [131].

#### 3.2 Epigenetic alterations

Alteration in epigenetic information is a primary driver of the aging process [132]. Increasing evidence suggests that epigenetic modifications play a role in the development of VA and AS [133].

#### 3.2.1 Noncoding RNAs

Noncoding RNAs (ncRNAs), including certain microRNAs (miRNAs), long noncoding RNAs (lncRNAs), and circular RNAs (circRNAs), are found to be essential in critical stages ranging from the progression of cell senescence and VA to the early creation of plaques and final maturity [134, 135].

MiR-181b can modulate the MAP3K3/MKK/MAPK signaling pathway for regulating EC apoptosis and senescence, which also play essential roles in cardiovascular inflammation [136, 137]. MiR-34a overexpression in SMCs induces SIRT1 downregulation for SMC senescence and the buildup of proinflammatory cytokines and adhesion molecules, thus accelerating the atherogenic process [138, 139]. In addition, miR-665 and miR-31-5p are upregulated in senescent SMCs, which regulate cell senescence and improve migratory ability [140, 141]. Furthermore, miR-200c is upregulated in response to ROS, leading to EC senescence and death by blocking ZEB1 [142]. Similarly, miR-217 is upregulated in ECs and reduces eNOS activator gene expression, resulting in NO-dependent endothelial dysfunction to accelerate arterial plaque formation [143]. Exosomal miRNAs, such as hsa-miR-155-5p, may alter the shape and functionality of vascular cells and VA [144, 145].

Researchers found that circANRIL can regulate pre-ribosomal RNA maturation, inducing cell apoptosis and inhibiting cell proliferation. When circANRIL expression is inhibited, rats appear to endothelial damage, oxidative stress, and inflammation [146]. CircGNAQ is relatively high in aortic tissues while continuously downregulated in aged ECs compared to the young ones, which may cause AS in aged mice [147]. In addition, research indicates that circACTA2 can compete with Cyclin-dependent kinase 4 (a protein kinase that regulates the cell cycle) to bind to ILF3 and decrease CDK4 mRNA stability and expression to induce Ang II-stimulated SMCs senescence [148]. circPVT1 decrease in senescent ECs, which operate as a competitive endogenous RNA to regulate CDK4 and its downstream genes by limiting miR-24-3p expression for delaying EC senescence [149].

LncRNAs were primarily found in ECs and showed a significant reduction in the presence of atherosclerotic plaques. For example, the loss of H19 caused the increased expression of p16 and p21 to decrease cell proliferation and aggravate cell senescence [150]. In addition, a study reported that microbial short RNAs (miRNAs) are enriched in LDLs for driving proinflammatory macrophage polarization and cytokine production by activating the RNA sensor Toll-like receptor 8, thus leading to inflammation and AS [151].

#### 3.2.2 DNA methylation and demethylation

A previous study reported that JunD promoter methylation reduces with age, resulting in the downregulation of free radical enzymes such as manganese superoxide dismutase, glutathione peroxidase-1, xanthine oxidase, and aldehyde dehydrogenase 2, which accelerates age-related endothelial dysfunction [152]. In addition, increased s-adenosyl-homocysteine (SAH) contributes to the demethylation of CpG islands in the NF-κB promoter region and enhances the expression of inflammatory-related secretory phenotypes in SMCs for cellular senescence and VA development [153].

### 3.3 Secretome changes and SASP

Although the proliferation of senescent cells is stopped, the metabolism remains active. The type of senescent cells and the senescence mechanisms may dictate the specific components of SASP [116, 154], which includes proinflammatory cytokines, growth factors, and extracellular matrix proteins. Some of these molecules act as aging signals communicated between cells to boost the increase of senescent cells and build up in the vasculature [155] (Figure. 3). SASP can induce AS by stimulating cell senescence. For example, the proinflammatory phenotype drives SMC senescence and immune cell recruitment to promote vascular plaque formation [131, 156, 157]. Similarly, senescent ECs may undergo a similar process [158, 159].

## 4. Vascular cell plasticity and AS

Vascular cells may exhibit heterogeneous changes with VA and AS. Senescent vascular cells have a predominance of gene sets associated with highly active senescence phenotypes and apoptotic processes, which serve as markers of aging [160, 161], allowing them to be identified by single-cell sequencing methods [155, 162, 163]. For example, SMCs transitioned to an intermediate cell state that could differentiate into macrophage-like and fibrochondrocyte-like cells in the AS process, which may also suffer phenotypic alterations at the lesion site during fibrous cap development [164, 165]. Furthermore, SMC’s plasticity and the ability to perform nonprofessional phagocytic functions maintain the inflammatory state and senescent condition in vessels [166]. Similarly, subpopulations of aortic ECs and their spatial heterogeneity change in response to high fat/salt/glucose conditions. This process may increase the expression of nitric oxide synthetase 3, endothelin 1, angiotensin-converting enzyme, and VCAM1 to develop AS [167]. Fibroblasts may differentiate into several fibroblast clusters in atherosclerotic tissue, which vary in gene expression and function [168]. Moreover, stromal cells’ differentiation declines in perivascular adipose tissue with age, resulting in vascular remodeling [169].

**Figure 4.**
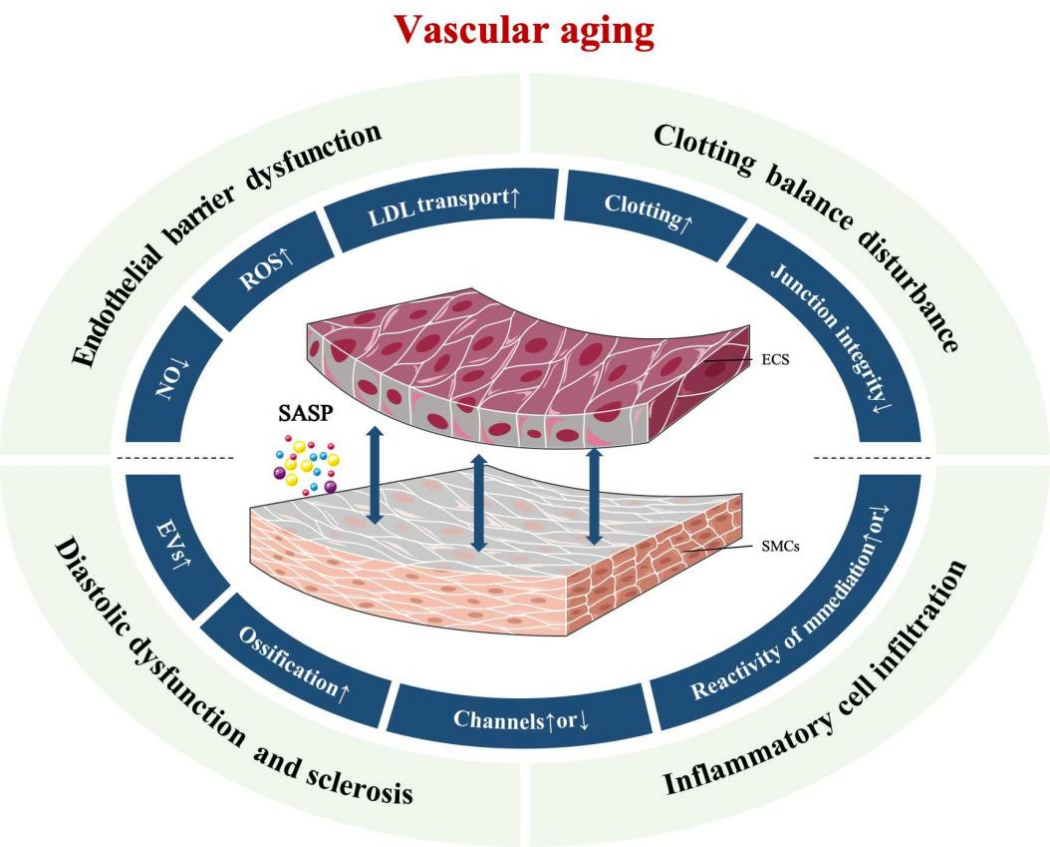
**Senescent ECs produce more ROS while reducing NO availability, along with increased EC permeability, the activation of coagulation, and inflammatory signals**. Senescent SMCs can activate osteogenic pathways, increase EV and inflammatory signal production, adjust cell membrane ion channels, and modify their sensitivity to vasoconstrictor and vasodilator mediators.

## 5. Effect of VA on AS

Biological aging provides the internal setting necessary for VA development. Senescent cells increase inside the vascular system, formulating VA and accelerating AS (Fig. 4).

Endothelial function and barrier integrity typically deteriorate due to EC senescence [170, 171]. For example, senescent ECs have been observed to suppress eNOS expression and activity [172, 173], resulting in decreased endothelial-dependent vasodilation [174]. Some connexins, such as cytosolic phospholipase A2 and claudin-5, are reduced in senescent cells, which impairs adherens and tight junction integrity [175]. Meanwhile, several oxidative stress signals such as ONOO-, O_2_^-^, and inflammatory factors like IL-6, IL-8, IL-1a, RANTES, as well as ICAM-1 are present for triggering interconnected pathways and cross-talk [176, 177]. For instance, ROS depletes NO, resulting in NO availability decline and the activation of inflammatory pathways [178]. Further, inflammatory signaling also has the potential to increase ROS generation and permeability and may interact to promote LDL and immune cell penetration into the subintima [171, 179]. In addition, senescent ECs may release more caveolin-1, an essential structural protein of caveolae that transports LDL entry into the intima through ECs [50, 180]. Furthermore, endothelial senescence raises thromboxane A2, von Willebrand factor, and plasminogen activator inhibitor-1 levels but decreases prostacyclin and thrombomodulin expression for platelet aggregation and assists in plaque formation [181]. Senescent SMCs increase secretory activity, impacting the formation and stability of atherosclerotic plaques (Fig. 4). For example, more extracellular vesicles (EVs) secreted by senescent SMC induces medin fibril formation for vascular amyloidosis and promote T-lymphocyte ( T cell) to generate IL-17, INF, and IL-10 while prompting monocytes to secrete TNF-α [182-184]. Meanwhile, senescent SMCs boost the expression of SASP components in plaque, such as the elevation of proinflammatory cytokines [185]. All of these inflammatory factors attract immune cells and help plaques. Further, senescent SMCs secrete proteases that degrade the extracellular matrix to increase plaque vulnerability [184, 186]. As SMC senescence, osteogenic pathways such as runt-related transcription factor-2 and alkaline phosphatase may be activated to induce vascular hardening and calcification processes [187, 188]. In addition, senescent SMC change susceptibility to vasoconstrictors and vasodilators, as well as ion channel density in the cell membrane, contribute to arterial wall stiffening [189].

**Figure 5.**
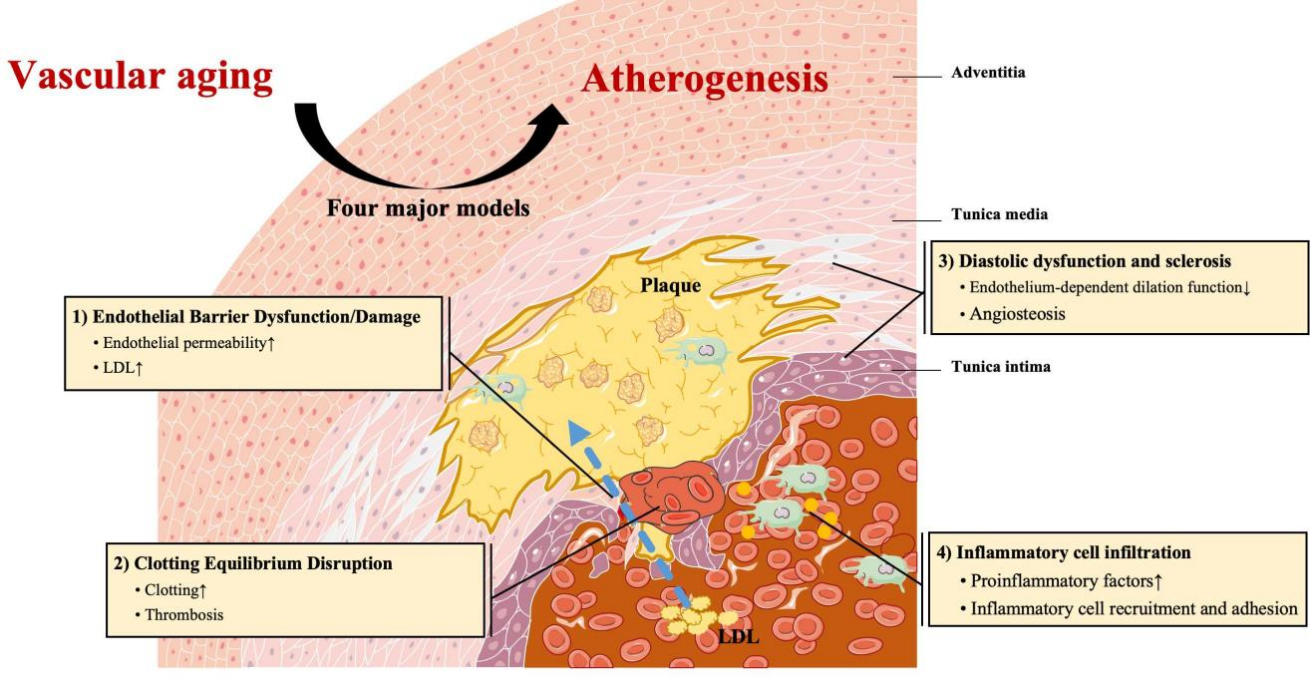
**Four major models of VA are critical in the promotion of AS: 1) Endothelial barrier dysfunction, 2) Clotting equilibrium disruption, 3) Diastolic dysfunction and sclerosis, and 4) Inflammatory cell infiltration**. Endothelium-dependent diastolic dysfunction and angiosteosis contribute to sclerosis. Impairment of endothelial function or barrier integrity can accelerate the deposition of LDLs in the intimal layer, potentially triggering platelet aggregation and thrombus formation. The vascular inflammatory response influences inflammatory cell recruitment and adhesion, promoting the formation of atherosclerotic plaques.

To summarize, four major models of VA are critically involved in the promotion of AS (Fig. 5). 1) Endothelial Barrier Dysfunction/Damage: The structure and function of the endothelium are prone to damage to deteriorate endothelial barrier function owing to VA, particularly at places with turbulent or unstable flow shear stress. LDL will travel easily through the endothelial barrier and accumulate inside the intimal layer to cause early lesions. 2) Clotting Equilibrium Disruption: Endothelial dysfunction upsets the delicate balance of local coagulation and anticoagulation mechanisms. The disruption can induce platelet dysfunction, aggregation, and the production of thrombi, further growing plaque. 3) Diastolic Dysfunction and Sclerosis: Endothelial diastolic dysfunction and smooth muscle calcification are both basics for sclerosis. 4) Inflammatory Cell Infiltration: Senescent vascular cells secrete proinflammatory factors that activate inflammatory pathways and recruit inflammatory cells, further infiltrating the intima and promoting lesion formation (Fig. 5).

## 6. Conclusion and prospects

VA is closely associated with AS, and the mechanism likely involves the senescence of eCs and SMCs that constitute the vasculature under the influence of various factors of biological aging. Senescent cells undergo phenotypic changes and accumulate in the vessel wall, leading to structural and functional damage, thereby promoting the occurrence and development of AS.

Recent research has identified twelve aging characteristics and eight cardiovascular aging markers, convoluted but closely related [190, 191]. Similar to the correlation between VA and AS, studies need to be investigated at different levels and dimensions, such as genetic, epigenetic, cellular, tissue, system, and function and incorporate multiple pathways and targets. Referring to the characteristics of aging vascular cells, eliminating or inducing senescent cell death, and inhibiting the expression of SASP-related molecules could be potential directions for therapies. While VA shows potential as a therapeutic target, clinical experience has shown that reducing the risk of age-related disorders demands a comprehensive management strategy incorporating various aspects, so more work needs to be optimized and integrated before these markers and mechanisms can be used to guide clinical decisions. On the other hand, disease management strategies often focus on intervening after disease onset and its progression. However, it is more important to address the fundamental factors that contribute to the development of the condition. Therefore, the next phase will focus on identifying clinical indicators of aging and developing methods to detect age-related diseases in their early stages.
